# Evaluation of the Fourier Frequency Spectrum Peaks of Milk Electrical Conductivity Signals as Indexes to Monitor the Dairy Goats’ Health Status by On-Line Sensors

**DOI:** 10.3390/s150820698

**Published:** 2015-08-21

**Authors:** Mauro Zaninelli, Alessandro Agazzi, Annamaria Costa, Francesco Maria Tangorra, Luciana Rossi, Giovanni Savoini

**Affiliations:** 1 Faculty of Agriculture, Università Telematica San Raffaele Roma, Via di Val Cannuta 247, 00166 Rome, Italy; 2 Department of Health, Animal Science and Food Safety (VESPA), Università degli Studi di Milano, Via Celoria 10, 20133 Milan, Italy; E-Mails: alessandro.agazzi@unimi.it (A.A.); annamaria.costa@unimi.it (A.C.); francesco.tangorra@unimi.it (F.M.T.); luciana.rossi@unimi.it (L.R.); giovanni.savoini@unimi.it (G.S.)

**Keywords:** frequency peaks, spectrum, electrical conductivity, Fast Fourier Transform, mastitis, dairy goats

## Abstract

The aim of this study is a further characterization of the electrical conductivity (EC) signal of goat milk, acquired on-line by EC sensors, to identify new indexes representative of the EC variations that can be observed during milking, when considering not healthy (NH) glands. Two foremilk gland samples from 42 Saanen goats, were collected for three consecutive weeks and for three different lactation stages (LS: 0–60 Days In Milking (DIM); 61–120 DIM; 121–180 DIM), for a total amount of 1512 samples. Bacteriological analyses and somatic cells counts (SCC) were used to define the health status of the glands. With negative bacteriological analyses and SCC < 1,000,000 cells/mL, glands were classified as healthy. When bacteriological analyses were positive or showed a SCC > 1,000,000 cells/mL, glands were classified as NH. For each milk EC signal, acquired on-line and for each gland considered, the Fourier frequency spectrum of the signal was calculated and three representative frequency peaks were identified. To evaluate data acquired a MIXED procedure was used considering the HS, LS and LS × HS as explanatory variables in the statistical model.Results showed that the studied frequency peaks had a significant relationship with the gland’s health status. Results also explained how the milk EC signals’ pattern change in case of NH glands. In fact, it is characterized by slower fluctuations (due to the lower frequencies of the peaks) and by an irregular trend (due to the higher amplitudes of all the main frequency peaks). Therefore, these frequency peaks could be used as new indexes to improve the performances of algorithms based on multivariate models which evaluate the health status of dairy goats through the use of gland milk EC sensors.

## 1. Introduction

In dairy farms, the detection of intramammary infections (IMI) by on-site techniques is a rapidly growing trend, favored by the time-consumption and costs that classic laboratory analytical techniques entail [1].

Many of these detection techniques use algorithms and sensors that analyze the electrical conductivity (EC) of milk [2,3,4,5,6,7,8,9,10,11,12,13,14,15,16,17,18]. The EC measures the ability of a solution to conduct an electric current between two electrodes and it is measured in milliSiemens per cm (mS/cm). In the milk there are anions and cations present in solution that give the fluid the ability to conduct an electric current [15,19]. The most important ones are: Na^+^, K^+^, and Cl^−^. The sodium pumps regulate the Na^+^ and K^+^ ions. These pumps are located on the baso-lateral membrane of the secretory cells and they pump Na^+^ into the extra-cellular fluid and K^+^ into cells while in the milk, the Na^+^ and K^+^ ions are transported passively across the apical membrane. Furthermore, a paracellular pathway is also present across the epithelium that allow Na^+^ and Cl^−^ to move into the milk and K^+^ and lactose to move into the extra-cellular fluid [15]. When an IMI is present, the EC of the milk increases [20,21] due to a higher concentration of Na^+^ and Cl^−^ in the milk. The destruction of tight junctions and of the active ion-pumping system is the main cause of these different concentrations [15]. In fact, Na^+^ and Cl^−^ leak into the lumen of the alveolus, and K^+^ and lactose move together out of the milk.

Although many factors, other than the health status of the mammary gland, can introduce some interpretation errors such as parity, lactation stage, and milk composition [22,23,24], the use of this parameter is a well consolidated practice with dairy cows [2,3,4,5,6,7,8,9,10,11,12,13,14,15,16,17,18] and many devices that evaluate the EC of milk measured in constant current (DC) or alternating current (AC—in a range of frequency between 50–70 kHz) [25] are available. On the contrary in dairy goats, the use of algorithms and sensors based on the milk EC and included in the milking systems in order to monitor online health status (HS) of the animals is not a widespread practice because of the low performances achieved to date [26,27,28,29].

The studies conducted on this technology (applied to dairy goats) are few and focus mainly on the relationships between the EC levels of milk and the HS of animals [28,29,30,31,32,33]. It is reported that milk EC signal can increase in infected goats [30] when major pathogens are the cause of the infection [32]. Significantly higher levels of milk EC can be found in different lactation stages [28]. The average value of the 20 highest measurements of the gland’s milk EC recorded online during a milking and in case of early lactation, may be used for the monitoring of the animals’ HS [28]. However, many of these studies suggest the use of more informative indexes built with a better knowledge of the relationship between the milk EC’ signal and the animals’ HS, along with improvements on the algorithms that analyze these data (based on multivariate models), as a possible way to reach good performance detection also in dairy goats [26,27,28,29,32,33,34].

In dairy cows, infected quarters may have a signal pattern of the milk EC during a milking with larger variations than those shown by healthy quarters. This effect has been highlighted by some authors [13] investigating the ability of three different models to detect mastitis based on the milk EC from udder quarters. Other authors [18], investigating the relationship between udder health status and different indexes based on the milk EC, have confirmed the same result showing that the statistical variance of all valid EC measures (σ^2^_EC_) increased from healthy to infected quarters with a greater difference in the case of clinical infected quarters. Also in dairy goats, similar results have been found. In a study conducted on a group of Saanen goats [28], observed during the entire lactation, our research group found that the index σ^2^_EC_ was greater in case of infected glands. However, in all these studies, the variations of the milk EC signal was evaluated through a general index as the statistical variance. Specific indexes able to characterize the milk EC signal patterns were not identified.

To this end, the spectral analysis of the milk EC signal could be a useful approach. This is a way to describe a signal from another point of view. All its characteristics in the time domain, as well as its pattern, can be described by its spectrum in the frequency domain. A spectrum can be obtained from a signal by specific mathematical operators, such as: the Fourier Transform (FT); the Discrete Fourier Transform (DFT), in case of signals discrete and made by a defined number of samples (N); or the Fast Fourier Transform (FFT), in case algorithms optimized and suitable for computer elaboration, are used. From these descriptions in the frequencies domain, different qualitative and quantitative indexes can be identified.

An example of this approach has been recently proposed by our research group [35]. Evaluating the spectra of the EC’ signal of dairy goat glands milk, it was discovered that the bandwidth length could be a possible index able to characterize the milk EC signal pattern. Results obtained have shown that mean values of the bandwidth length increased in the case of not healthy (NH) glands. Furthermore, a description was given on how the EC signal pattern changed in the time domain since an increase in the bandwidth length generally involves a signal pattern characterized by faster oscillations, most likely with larger amplitudes. However, the bandwidth length does not express all the information content that the Fourier frequency spectrum of a signal can provide. Other indexes could be added in order to reach a more detailed description of the signal pattern in the time domain. For example, often a spectrum has peaks of bigger amplitude. These peaks (in some cases called harmonics) characterize the signal in the frequency domain and give a further description of the main characteristics of the signal pattern also in the time domain. Through these peaks, or subsets of them, new indexes could be developed with the future target to improve the detection performances of algorithms that use multivariate models to evaluate the HS of dairy goats by the use of online gland milk EC sensors.

The aim of this study was to further describe the milk EC signal pattern in the time domain, and in the case of NH glands, through new qualitative and quantitative indexes based on the frequency peaks of the milk EC signal spectrums.

## 2. Experimental Section

### 2.1. Animals and Farm Management

The experiment was carried out at the Experimental Farm of the University of Milan, Italy. Forty-two second-parity Saanen goats, 10 ± 5 days after delivery, were randomly selected for the trial. Animals were fed twice a day with a common lactating basal diet for the whole experimental period on the basis of their nutritional requirements NRC (2007). Goats were milked twice a day at 7:00 a.m. and 5:00 p.m. with a low-line milking parlor that included: self-locking gates, 32 milking units equally distributed on two platforms. Milking parameters set-up for the milking system were: a rate of 90 pulsations per minute, a machine vacuum level of 40 kPa and had a pulsation ratio of 60%.

### 2.2. Experimental Design, Milk Sample Collection and Analyses

The experiment was carried out for six months with the farm being visited a total of nine times. The sampling frequency was weekly and repeated three times for each lactation stage that was evaluated (0–60 Days In Milk [DIM]; 61–120 DIM; 121–180 DIM). For each farm visit, the collection of milk samples was done during the morning milking after the teat disinfection (with chlorhexidine-moistened towels) and the discharging of the first milk streams. From each mammary gland of the animals’ trial group, two individual milk samples were taken.

A total amount of 1512 milk samples were collected during the trial. From these samples, 756 were used for bacteriological analysis (*i.e.*, one for each gland, week and LS considered) according to the International Dairy Federation standard method (FIL-IDF, 1981) while the other 756 were analyzed for somatic cell counts (SCC) using a Bentley SomacountTM 500 analyzer (Bentley Instruments Inc., Chaska, MN, USA) and following the FIL-IDF (1995) recommendations.

According to the results of microbiological tests and SCC, samples were classified as healthy glands when somatic cell counts were less than 1,000,000 cells/mL and pathogenic microorganisms were absent and deemed NH mammary glands when bacteriological analyses were positive for IMI or SCC were more than 1,000,000 cells/mL for non-physiological causes [29,33] (as for example estrus or the end of lactation). When milk samples were collected, milk EC signals were also measured and stored by the data acquisition system. Milk EC signals acquired were from each mammary gland of the animals’ trial group.

### 2.3. Milk Electrical Conductivity Measures and Data Acquisition System

**Figure 1:**
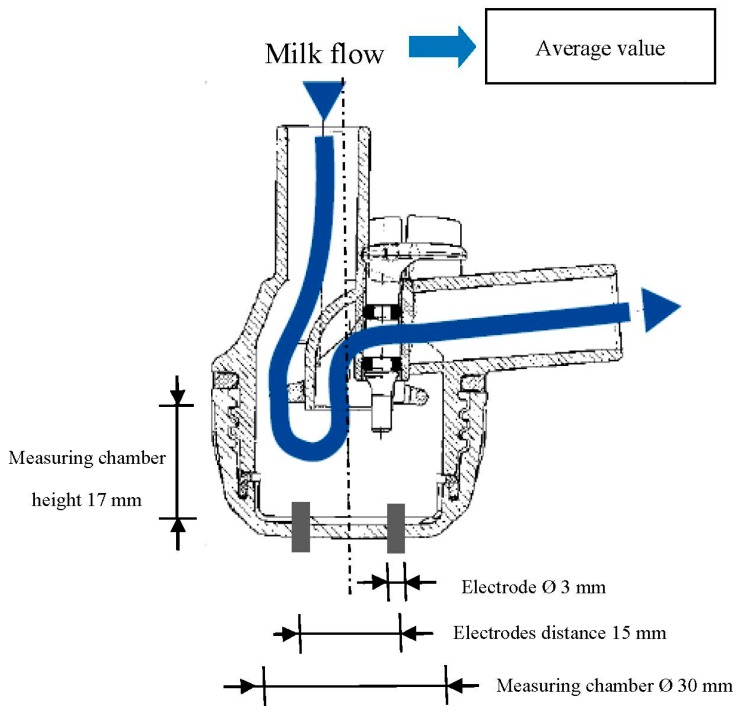
Dimensions of the EC sensor head.

**Figure 2:**
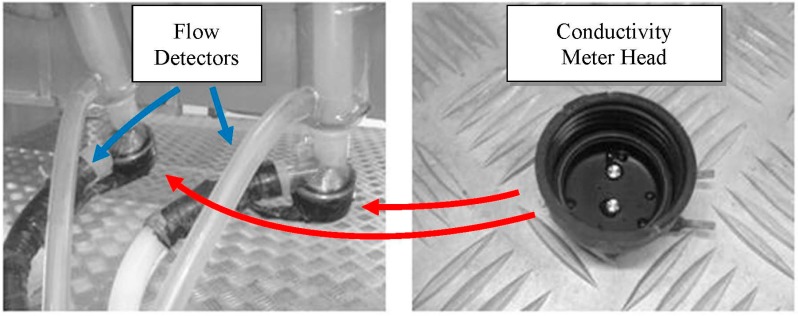
View of prepared sensor heads. In the left picture, the positions of the conductivity meter heads and of the flow detectors, included in each experimental milking cluster, are also highlighted.

Four experimental milking clusters were used to measure the milk EC from each gland. These experimental milking clusters were developed through modifying commercial milking units (Vanguard, Interpuls S.p.A., Albinea (RE), Italy) [26,27,28,35]. Each experimental milking cluster included two EC sensors. Each EC sensor was made by a couple of stainless cylindrical electrodes (Figure 1) placed at the base of each individual milking claw (Figure 2). This hardware allowed the measuring of the specific EC of milk (in milliSiemens—mS/cm) while it was flowing from the gland to the milk line. Furthermore, a flow detector was placed inside each short milk tube of the milking cluster (Figure 2). It was made by an additional couple of cylindrical stainless electrodes that, measuring a signal proportional to the filling level of the short milk tube, allowed to monitor the beginning and the end of each milking and to avoid or correct possible data error due to the presence of milk residue in the milking claws.

**Figure 3:**
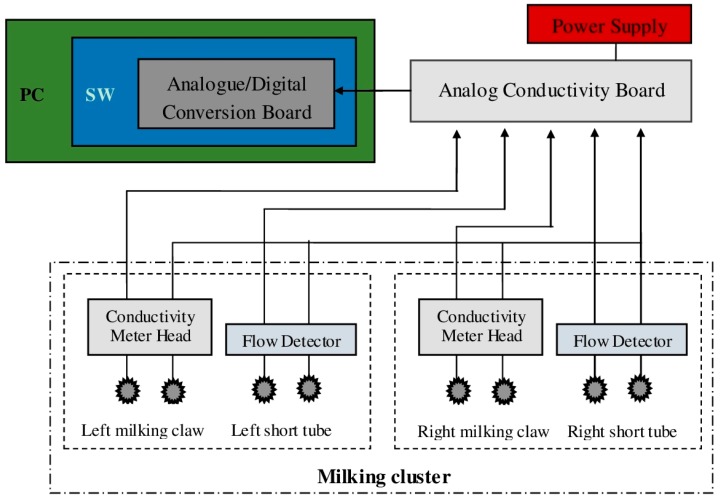
Block schema of the recording system. In the schema only one “analog conductivity board” and milking cluster is reported to simplify the reading of the figure.

All the electrical signals from the milking clusters were evaluated by four analog conductivity boards (output range 0–10 V, accuracy ±0.1%) placed in a separate room next to the milking parlor, and acquired from an analogue/digital conversion board installed in a PC (DAQCard AI-16E-4, National Instruments, Austin, TX, USA—with a resolution of 12 bit and a total sampling rate of 250 kS/s). Furthermore, through customized software application developed using LabVIEW 8.02 (National Instruments), acquired data were sampled with a rate of 1 Hz and stored as .txt files using: the goat ID farm number, date and time to name each file. A complete block schematic of the whole recording system is provided in Figure 3.

Before the start of the experiment, laboratory tests were carried out in order to check the effects of different milk flow rates on the measurements made by the EC sensors. A solution of water and chlorine-based detergent for milking machine was used as fluid test. The detergent was added to the water to increase its EC up to 6 mS/cm. Two EC sensors (included in the same milking cluster) were tested at constant liquid flow rates—from 0.2 L/min to 1.0 L/min in incremental steps of 0.2 L/min—using a suitable artificial udder equipped with a flow regulator. Ten repetitions were made for each flow rate investigated, for a total amount of 100 readings (*i.e.*, 10 repetitions per five flow rates per two sensors or milking claws). For each repetition performed, approximately 5 L of fluid test passed through the milking cluster and the electrical signals measured from the sensors were stored by the recording system. As following steps: (1) electrical mean values of each reading were calculated; (2) electrical mean values for each flow rate tested were calculated; (3) the overall measurement accuracy was estimated considering the differences between the electrical mean value measured at 0.6 L/min and electrical mean values of the other flow rates tested. The flow rate of 0.6 L/min was taken as reference because considered as the average milking flow rate expected in the following filed tests.

Similar laboratory tests were also performed in order check the linearity of the EC sensors and to make their calibrations. The same kind of fluid test was used, but in this case, the detergent was added to the water to increase its EC from 4 mS/cm up to 12 mS/cm, by incremental steps of 2 mS/cm. All the EC sensors were tested at a constant liquid flow rate of 0.6 L/min. Ten repetitions for each EC level and for each experimental milking cluster were made for a total of 400 readings (*i.e.*, 10 repetitions per 5 EC levels per eight sensors or milking claws). Also in these cases, for each repetition performed approximately 5 L of fluid test passed through the milking cluster and the electrical signals measured from the sensors were stored by the recording system. As following steps: (1) electrical mean values of each reading were calculated; (2) electrical mean values for each EC level tested were calculated; (3) on the resulting data, a linear regression was performed for each sensor tested. At the end of these tests, obtained results allowed to set-up each EC sensor.

### 2.4. Fourier Frequency Spectrum Calculation

**Figure 4:**
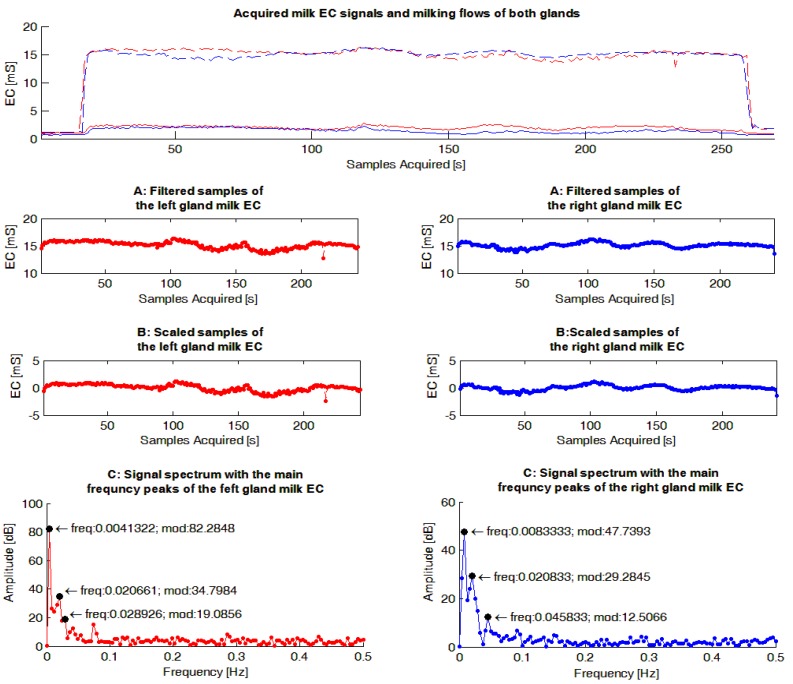
Example of gauges obtained from the milk electrical conductivity (EC) signals acquired within a milking. The graphs in red show data concerning the left gland while those in blue are reported data for the right gland. In the upper graph the measured EC signals of milk acquired during a milking from each gland are reported. In same graph the milk flows recorded by the experimental milking cluster used during the trial are also shown. The following graphs report on: (1) the sequences without the signal samples related to the start and the end of milking (“A: Filtered samples of gland milk EC”); (2) the sequences where the mean value of each sequence have been subtracted to each signal sample acquired (“B: Scaled samples of gland milk EC”); (3) the spectrums obtained, applying the Fast Fourier Transform to the previous sequences of signal samples, and the three main frequency peaks identified for each Fourier frequency spectrum (“C: Signal spectrum with the main frequency peaks of the gland milk EC”).

The milk EC signals were evaluated by a dedicated Matlab routine (The Mathworks, Natick, MA, USA). The main steps performed by the software routine (Figure 4) were the following: (1) samples related to the start and the end of a milking were filtered from the sequence; (2) the mean value of the resulting signal was calculated and subtracted to each sample of the sequence in order to have a Fourier frequency spectrum with a null peak at the frequency of zero, and consequently, a scaled graph in the frequency domain useful in identifying the most important peaks; (3) on the resulting sequence, the FFT was calculated.

As a following step, the software routine was set-up to identify the three highest frequency peaks of each Fourier frequency spectrum calculated to use in the statistical analyses (FFT_P_1,2,3_), and for each of them, identify the corresponding frequency and amplitude (magnitude). This set-up was chosen in order to consider the most important information included in each spectrum through a reasonable number of parameters.

### 2.5. Statistical Analyses

In order to investigate data acquired during the trial, the Shapiro-Wilk test was used to confirm the normal distribution of all the variables studied. A MIXED procedure (SPSS Statistics, version 21, IBM SPSS, Armonk, NY, USA) was used to evaluate the association between SCC, EC (log transformed in order to normalize their distributions) and the explanatory variables. The statistical model used is the following:

$$Y_{ijkl}=\mu +\;HS_{i}+\;LS_{j}+\;HS*LS_{ij}+\;{\text{δ}}_{k}\left({\text{ε}}_{l}\right)+{\text{ε}}_{l}+e_{ijkl}$$

where: Y is the SCC or EC, μ is the mean, HS_i_ is the effect of health status (i = 0–1; 0 = healthy; 1 = NH), LS_j_ is the effect of lactation stage (j = 1–3; 1 = 0–60 Days In Milking; 2 = 61–120 DIM; 3 = 121–180 DIM), HS*LS_ij_ is the interaction between health status and lactation stage, δ_k_(ɛ_l_) is the random effect of the gland (k = 1–2; 1 = left, 2 = right) nested to the goat (l = 1–42), ɛ_l_ is the random effect of the goat (l = 1–42) and e_ijkl_ is the residual error [36]. Furthermore, an unstructured covariance structure was used to account for the repeated measurements [29,33].

As a following step, in order to investigate the milk EC signal spectrums, another MIXED procedure was used. The association between the frequency peaks investigated (FFT_P_n_) and the explanatory variables were studied. The statistical model used was the same as above described. In the model, the frequency peaks—in terms of frequency and amplitude of each peak—were set-up as dependent variables.

As a final step, in order to show which parameter would be the most useful index to detect the health status of dairy goats for each frequency peak evaluated—always in terms of frequency and amplitude of each peak—sensitivity and specificity were calculated. In this context, sensitivity represents the percentage of glands correctly identified as NH respect to all the cases of milk samples classified as belonging to NH glands:

Sensitivity = True Positive/(True Positive + False Negative)*100

The specificity indicates the percentage of glands correctly identified as healthy in respect to all the cases of milk samples classified as belonging to healthy glands:

Specificity = True Negative/(False Positive + True Negative)*100

In order to complete the comparison between the peaks studied, a sensitivity of 80% was chosen because it is considered as the gold standard of human observation [37]—although this threshold can be affected by variables such as the skills of the milker and the severity of the case. Accordingly, for each frequency peak evaluated, specific cut-off levels were determined in order to reach a sensitivity of at least 80%. The resulting pairs of sensitivity and specificity were calculated and considered the level of accuracy reached by each parameter investigated.

## 3. Results

Laboratory tests carried out at different flow rates showed an overall sensor accuracy of 0.94%, with no relevant differences between the two EC sensors evaluated. Furthermore, linear trends within the range of the EC levels investigated were confirmed for all the EC sensors tested. In the Table 1 the parameters of the linear regressions performed are reported.

**Table 1:** Parameters of the linear regressions performed on the EC sensors used in the experiment and evaluated at different EC levels—from 4 mS/cm up to 12 mS/cm, by incremental steps of 2 mS/cm.

Sensor	Angular Coefficient	R^2^
*1*	2.36	0.98
*2*	2.17	0.99
*3*	2.22	0.97
*4*	2.15	0.98
*5*	2.30	0.99
*6*	2.11	0.97
*7*	2.19	0.98
*8*	2.15	0.95

After the microbiological evaluation of milk, seven samples resulted contaminated. The prevalence of positive samples was 68.4% (*n* = 517, Table 2) with Coagulase-negative *Staphylococcus* as the most prevalent mastitis agent (62.6%, Table 2). After the count of somatic cells in the milk samples, the resulting prevalence of glands with SCC > 1,000,000 and without pathogenic microorganisms was 4.8% (*n* = 36, Table 3). No cases of SCC > 1,000,000 due to physiological causes were observed. The overall prevalence of samples from NH glands was 73.8% (Table 3) and no cases of clinical mastitis were observed.

**Table 2:** Distribution of pathogenic microorganisms found in infected mammary glands. Overall means and standard errors, of SCC (log) and EC (mS/cm) of gland milk samples, according to each of the groups of pathogenic microorganisms found were also reported.

Isolated Bacterial Strains	*n*	%	SCC Mean ± S.E. (logSCC)	EC Mean ± S.E. (mS/cm)
*Coagulase-negative Staphylococcus (CNS)*	473	62.6	5.44 ± 0.02	14.37 ± 0.27
*Escherichia coli*	3	0.4	5.32 ± 0.48	11.90 ± 2.45
*Streptococcus* spp.	18	2.4	5.34 ± 0.09	11.24 ± 0.51
*Lactose-negative bacteria*	13	1.7	5.55 ± 0.10	15.02 ± 0.61
*Pseudomonas* spp.	10	1.3	5.53 ± 0.09	15.77 ± 0.36
*Contaminated*	7	0.9	-	-
*BC negative*	232	30.7	5.38 ± 0.03	12.56 ± 0.03

**Table 3:** Distribution of mammary glands for each health status considered. Samples were classified as collected from healthy glands when somatic cell counts were less than 1,000,000 cells/mL and pathogens were absent, and classified as collected from NH mammary glands when bacteriological analyses were positive for IMI or SCC were more than 1,000,000 cells/mL.

Health Status of Glands	*n*	%	Samples with Positive Bacteriological Analyses and SCC < 1,000,000 (cells/mL)		Samples with Positive Bacteriological Analyses and SCC > 1,000,000 (cells/mL)		Samples with with Negative Bacteriological Analyses and SCC > 1,000,000 (cells/mL)	
			n	%	n	%	n	%
**Healthy**	196	26.2	-	-	-	-	-	-
**Not healthy**	553	73.8	434	57.9	83	11.1	36	4.8

Not healthy glands showed a higher significant mean value of SCC (5.20 ± 0.04 [logSCC] *vs.* 5.49 ± 0.03, Table 4). Furthermore, the second and third lactation stage showed significantly increased levels of SSC (5.19 ± 0.04 [logSCC] *vs.* 5.46 ± 0.04 and 5.59 ± 0.04, Table 4) if compared with the first lactation stage. However, the interaction between the HS and LS was not significant.

**Table 4:** Overall means and standard errors of SCC (log) of gland milk samples according to HS and lactation stages.

Health Status of Glands:	Days in Milking:			
	*0–60* Mean ± S.E. (logSCC)	*61–120* Mean ± S.E. (logSCC)	*121–180* Mean ± S.E. (logSCC)	*0–180* Mean ± S.E. (logSCC)
**Healthy**	5.10 ± 0.05	5.30 ± 0.10	5.52 ± 0.10	5.20 ^A^ ± 0.04
**Not healthy**	5.28 ± 0.06	5.50 ± 0.04	5.61 ± 0.04	5.49 ^B^ ± 0.03
**All**	5.19 ^X^ ± 0.04	5.46 ^Y^ ± 0.04	5.59 ^Y^ ± 0.04	5.41 ± 0.02

^A,B^ means in the same column with different uppercase superscripts differ significantly (*p* < 0.01); ^X,Y^ means in the same row with different uppercase superscripts differ significantly (*p* < 0.01).

Not healthy glands also showed significantly higher values of milk EC (12.27 ± 0.17 [mS/cm] *vs.* 14.27 ± 0.10, Table 5). Furthermore, a significantly lower mean value of milk EC was observed in the first stage when compared with the other lactation stages (11.13 ± 0.08 [mS/cm] *vs.* 15.12 ± 0.04 and 14.87 ± 0.03). Also for these cases, the interaction between the HS and LS was not significant.

**Table 5:** Overall means and standard errors of EC (mS/cm) of gland milk samples according to HS and lactation stages.

Health Status of Glands:	Days in Milking:			
	*0–60* Mean ± S.E. (mS/cm)	*61–120* Mean ± S.E. (mS/cm)	*121–180* Mean ± S.E. (mS/cm)	*0–180* Mean ± S.E. (mS/cm)
**Healthy**	10.95 ± 0.09	14.86 ± 0.34	14.53 ± 0.39	12.27 ^A^ ± 0.17
**Not healthy**	11.32 ± 0.11	15.39 ± 0.13	15.21 ± 0.11	14.27 ^B^ ± 0.10
**All**	11.13 ^X^ ± 0.08	15.12 ^Y^ ± 0.04	14.87 ^Y^ ± 0.03	13.75 ± 0.09

^A,B^ means in the same column with different uppercase superscripts differ significantly (*p* < 0.01); ^X,Y^ means in the same row with different uppercase superscripts differ significantly (*p* < 0.01).

About the spectrums evaluated and the relative frequency peaks investigated, data showed a normal distribution for the frequencies and amplitudes of the peaks. Mean values of FFT_P_1_ frequency were significantly lower in NH glands (13.98 ± 0.82 × 10^−3^ (Hz) *vs.* 9.94 ± 0.31 × 10^−3^, Table 6) and significantly lower in different lactation stages (13.74 ± 0.64 × 10^−3^ (Hz), 11.05 ± 0.55 × 10^−3^ and 8.28 ± 0.39 × 10^−3^). Furthermore, the peak’s mean amplitude was significantly higher in NH glands (34.01 ± 2.13 (dB) *vs.* 48.58 ± 1.53—Table 7) and during the progress of lactation (29.26 ± 1.39 (dB), 49.87 ± 2.37 and 56.05 ± 2.40). However, the interaction between the HS and LS was not significant for both the mean values of frequency and amplitude of the peak. With the peak FFT_P_2_, the mean frequency values showed a significant trend between different HS (62.02 ± 5.09 × 10^−3^ (Hz) *vs.* 46.23 ± 1.99 × 10^−3^—Table 8) and it reported significantly lower levels during the progress of lactation (60.77 ± 4.40 × 10^−3^ (Hz), 48.61 ± 3.50 × 10^−3^ and 41.53 ± 1.95 × 10^−3^). Furthermore, the mean peak amplitude values showed a significant increase in NH glands (12.56 ± 0.74 (dB) *vs.* 18.47 ± 0.53—Table 9) and significantly higher levels during the progress of lactation were observed (10.76 ± 0.53 (dB), 18.47 ± 0.80 and 21.76 ± 0.78). This peak also showed that the interaction between the HS and LS was not significant. Ultimately, the third peak evaluated (FFT_P_3_) showed similar results as of those obtained by the other studied peaks. Significantly lower means of frequency were observed between different statuses of the mammary glands (111.01 ± 7.16 × 10^−3^ (Hz) *vs.* 81.21 ± 3.15 × 10^−3^, respectively for healthy or NH glands—Table 10) and lactation stages (112.27 ± 6.07 × 10^−3^ (Hz), 89.05 ± 5.72 × 10^−3^ and 66.31 ± 3.30 × 10^−3^), and significantly higher mean values of amplitude were measured in NH glands (7.65 ± 0.46 (dB) *vs.* 11.57 ± 0.34—Table 11) and in different lactation stages (6.29 ± 0.30 (dB), 11.10 ± 0.49 and 14.28 ± 0.52). Interaction between the HS and LS was also in this case not significant.

**Table 6:** Overall means and standard errors of the frequency of peak FFT_P_1_, according to health status and lactation stages.

Health Status of Glands:	Days in Milking:			
	*0–60* Mean ± S.E. (Hz)	*61–120* Mean ± S.E. (Hz)	*121–180* Mean ± S.E. (Hz)	*0–180* Mean ± S.E. (Hz)
**Healthy**	14.88 ± 1.05 × 10^−3^	13.00 ± 1.55 × 10^−3^	11.56 ± 2.1 × 10^−3^	13.98 ^A^ ± 0.82 × 10^−3^
**Not healthy**	12.71 ± 0.74 × 10^−3^	10.62 ± 0.57 × 10^−3^	7.85 ± 0.34 × 10^−3^	9.94 ^B^ ± 0.31 × 10^−3^
**All**	13.74 ^X,x^ ± 0.64 × 10^−3^	11.05 ^y^ ± 0.55 × 10^−3^	8.28 ^Y^ ± 0.39 × 10^−3^	10.99 ± 0.32 × 10^−3^

^A,B^ means in the same column, with different uppercase superscripts differ significantly (*p* < 0.01); ^X,Y^ means in the same, row with different uppercase superscripts differ significantly (*p* < 0.01); ^x,y^ means in the some row, with different lowercase superscripts differ significantly (*p* < 0.05).

**Table 7:** Overall means and standard errors of the amplitude of peak FFT_P_1_, according to health status and lactation stages.

Health Status of Glands:	Days in Milking:			
	*0–60* Mean ± S.E. (dB)	*61–120* Mean ± S.E. (dB)	*121–180* Mean ± S.E. (dB)	*0–180* Mean ± S.E. (dB)
**Healthy**	27.40 ± 1.92	42.32 ± 4.90	50.63 ± 8.08	34.01 ^a^ ± 2.13
**Not healthy**	30.93 ± 1.43	51.52 ± 2.67	56.77 ± 2.51	48.58 ^b^ ± 1.53
**All**	29.26 ^X,x^ ± 1.39	49.87 ^y^ ± 2.37	56.05 ^Y^ ± 2.40	44.76 ± 1.28

^a,b^ means in the same column with different lowercase superscripts differ significantly (*p* < 0.05); ^X,Y^ means in the same, row with different uppercase superscripts differ significantly (*p* < 0.01); ^x,y^ means in the some row, with different lowercase superscripts differ significantly (*p* < 0.05).

**Table 8:** Overall means and standard errors of the frequency of peak FFT_P_2_, according to health status and lactation stages.

Health Status of Glands:	Days in Milking:			
	*0–60* Mean ± S.E. (Hz)	*61–120* Mean ± S.E. (Hz)	*121–180* Mean ± S.E. (Hz)	*0–180* Mean ± S.E. (Hz)
**Healthy**	66.23 ± 7.26 × 10^−3^	61.29 ± 8.90 × 10^−3^	46.13 ± 5.35 × 10^−3^	62.02 ^a^ ± 5.09 × 10^−3^
**Not healthy**	55.84 ± 5.21 × 10^−3^	45.83 ± 3.78 × 10^−3^	40.93 ± 2.09 × 10^−3^	46.23 ^b^ ± 1.99 × 10^−3^
**All**	60.77 ^X^ ± 4.40 × 10^−3^	48.61 ± 3.50 × 10^−3^	41.53 ^Y^ ± 1.95 × 10^−3^	50.36 ± 2.00 × 10^−3^

^a,b^ means in the same column with different lowercase superscripts differ significantly (*p* < 0.05); ^X,Y^ means in the same, row with different uppercase superscripts differ significantly (*p* < 0.01).

**Table 9:** Overall means and standard errors of the amplitude of peak FFT_P_2_, according to health status and lactation stages.

Health Status of Glands:	Days in Milking:			
	*0–60* Mean ± S.E. (dB)	*61–120* Mean ± S.E. (dB)	*121–180* Mean ± S.E. (dB)	*0–180* Mean ± S.E. (dB)
**Healthy**	10.33 ± 0.73	14.14 ± 1.43	19.61 ± 2.65	12.56 ^A^ ± 0.74
**Not healthy**	11.15 ± 0.76	19.42 ± 0.91	22.04 ± 0.82	18.47 ^B^ ± 0.53
**All**	10.76 ^X^ ± 0.53	18.47 ^Y^ ± 0.80	21.76 ^Z^ ± 0.78	16.92 ± 0.44

^A,B^ means in the same column, with different uppercase superscripts differ significantly (*p* < 0.01); ^X,Y,Z^ means in the same, row with different uppercase superscripts differ significantly (*p* < 0.01).

**Table 10:** Overall means and standard errors of the frequency of peak FFT_P_3_, according to health statuses and lactation stages.

Health Status of Glands:	Days in Milking:			
	*0–60* Mean ± S.E. (Hz)	*61–120* Mean ± S.E. (Hz)	*121–180* Mean ± S.E. (Hz)	*0–180* Mean ± S.E. (Hz)
**Healthy**	118.85 ± 9.53 × 10^−3^	111.52 ± 13.95 × 10^−3^	79.30 ± 14.52 × 10^−3^	111.01^a^ ± 7.16 × 10^−3^
**Not healthy**	106.35 ± 7.71 × 10^−3^	84.13 ± 6.23 × 10^−3^	64.60 ± 3.20 × 10^−3^	81.21^b^ ± 3.15 × 10^−3^
**All**	112.27 ^X^ ± 6.07 × 10^−3^	89.05^x^ ± 5.72 × 10^−3^	66.31^Y,y^ ± 3.30 × 10^−3^	89.01 ± 3.02 × 10^−3^

^a,b^ means in the same column with different lowercase superscripts differ significantly (*p* < 0.05); ^X,Y^ means in the same, row with different uppercase superscripts differ significantly (*p* < 0.01); ^x,y^ means in the some row, with different lowercase superscripts differ significantly (*p* < 0.05).

**Table 11:** Overall means and standard errors of the amplitude of peak FFT_P_3_, according to health statuses and lactation stages.

Health Status of Glands:	Days in Milking:			
	*0–60* Mean ± S.E. (dB)	*61–120* Mean ± S.E. (dB)	*121–180* Mean ± S.E. (dB)	*0–180* Mean ± S.E. (dB)
**Healthy**	5.88 ± 0.37	9.19 ± 0.99	12.85 ± 1.85	7.65 ^a^ ± 0.46
**Not healthy**	6.65 ± 0.47	11.52 ± 0.55	14.46 ± 0.54	11.57 ^b^ ± 0.34
**All**	6.29 ^X^ ± 0.30	11.10 ^Y^ ± 0.49	14.28 ^Z^ ± 0.52	10.54 ± 0.29

^a,b^ means in the same column with different lowercase superscripts differ significantly (*p* < 0.05); ^X,Y,Z^ means in the same, row with different uppercase superscripts differ significantly (*p* < 0.01).

Lastly, the health status detection accuracy reached by the studied frequency peaks was investigated. Specific cut-off levels were determined for each frequency peak in order to reach a sensitivity of at least 80%. Consequently, pairs of sensitivity and specificity were calculated. Results showed that the amplitude value, of the frequency peak FFT_P_2_, reached the best accuracy (specificity = 42.9% and sensitivity = 80.1%, Table 12) with a cut-off level equal to 16.05 dB followed by the frequency value of the frequency peak FFT_P_1_ (specificity = 39.8% and sensitivity = 80.1%) with a cut-off level equal to 12.00 × 10^−3^ Hz.

**Table 12:** Accuracy of the studied frequency peaks reported in terms of sensitivity and specificity of each peak at specific cut-off levels.

Peaks (FFT_P_n_)	Characteristics Frequency (Hz)—Amplitude (dB)	Cut-off Level (Hz–dB)	Specificity (%)	Sensitivity (%)
FFT_P_1_	Frequency	12.00 × 10^−3^	39.8	80.1
	Amplitude	40.94	36.7	80.1
FFT_P_2_	Frequency	60.50 × 10^−3^	25.0	80.1
	Amplitude	16.05	42.9	80.1
FFT_P_3_	Frequency	102.00 × 10^−3^	32.7	80.1
	Amplitude	8.81	38.3	80.3

## 4. Discussion

Laboratory tests showed that the specific design of the milking cluster selected for the experiment allowed us to isolate a defined quantity of milk in all the range of flow rates investigated. Consequently, the overall accuracy achieved by the sensors was fine. The EC sensors also showed a good linearity in the range of the EC levels studied. Therefore, the calibrations of the EC sensors were possible through the angular coefficients found and the measurements of the specific EC of milk available for the following field tests carried out.

The microbiological analyses showed a high prevalence of bacteriological positive samples (69.0%). The persistence of subclinical IMI, during lactation, is variable according to the causative pathogen. However, the persistence of subclinical IMI is generally high if Staphylococci (as it was in this study—CNS: 91.5%) is the major pathogen because it has the potential to become a chronic infection [38]. Somatic cell count was significantly higher in milk samples from NH glands and showed to significant increase between the first, the second, and third lactation stage. Other authors found the highest values of SCC for infected glands [29,32] and a significant increase of the average value of SCC during lactation [28,33]. Also the mean values of milk EC showed to be significantly higher in NH glands and increased during the progress of lactation. Similar results were reported by other authors. In cases of infected glands a significant increase of milk EC was observed [32,33] and with the progress of lactation, higher levels of milk EC were measured [28].

Mean values of milk EC, found in the present study, were higher than those reported by other authors [1,29,32,33,34,39]. These different results may be explained by the characteristics of the measuring system used. It included four experimental milking clusters developed in order to measure online the gland’s milk EC signal without affecting the flow of milk from glands and the vacuum of the milking system. Having these targets, the lowest number of components was added to the commercial milking clusters. Thus, no temperature sensors were included, and consequently, no temperature adjustments were possible during the recordings of the milk EC data. Effects related to the milking procedures could have also conditioned the milk EC readings [39]. For example, it is possible that the average quantity of milk, in the measurement chamber of the EC sensors, was not the same as supposed by the calibration procedure. However, the spectral analyses performed were not affected by all these aspects because, during the elaboration of the data acquired, the mean value of each milk EC signal was calculated and subtracted for each sample of the corresponding sequence recorded.

In order to investigate the milk EC signals’ spectrum, the relationship between the studied frequency peaks (FFT_Pn) and the glands HS were studied. All the peaks showed significant lower values of frequency in case of NH glands and significantly increased levels of amplitude when NH glands were considered. These results described how the milk EC signals’ spectrum changed in case of infected glands. The results highlighted that the EC signals’ pattern of milk from infected gland—in the time domain—were characterized by an increased internal variation (since the amplitude of the peaks were generally higher in NH cases). Similar results were also reported by other authors analyzing the standard deviation (σ^2^_EC_) of the milk’s EC signal. In a study conducted on a group cows, some authors [13] found higher values of σ^2^_EC_, in the case of mastitis (0.58 to 0.71—depending on the specific minute of milking evaluated), than in the case of healthy quarters (0.16 to 0.34). In another study on cows, other authors [18] confirmed that σ^2^_EC_ increased between healthy quarters and infected quarters. Furthermore, they found that the difference between clinical infected quarters and healthy quarters was greater than the difference between subclinical infected quarters and healthy quarters. Finally, also our research group, in a previous study that involved a group of goats monitored for the whole lactation [28], reported that σ^2^_EC_ was grater in infected glands during the second and third lactation stage (0.14 *vs.* 0.16 and 0.21 *vs.* 0.22).

A general index like the statistical variance is not able to characterize the milk EC signal pattern in the time domain. For this purpose, the spectral analysis of the milk EC signal can be a useful approach. A recent study [35] on the milk EC signals collected from dairy goats, conducted by our research group, shows that the mean value of the bandwidth length increased in the case of NH glands (from 0.24 Hz to 0.29 Hz). This result confirms that in the case of infected glands an increased signal variance is expected. However, this information is not enough to characterize the milk EC signals’ pattern in a unique way because an increase of the bandwidth length can be due to a vast number of changes in frequencies and/or amplitudes of the main peaks that characterize the spectrum. On the contrary, the results obtained in the study gave more detailed information on how the milk EC signal patterns changed in the time domain. In the case of infected glands, results showed that the signals’ pattern is generally characterized by slower fluctuations (due to the lower frequencies of the peaks) and by a more irregular trend (due to the higher amplitudes of all the main frequency peaks).

The frequency peaks that were investigated also showed significant results during lactation. All the peaks (FFT_Pn) showed lower mean values of frequency between different lactation stages and higher mean values of amplitude during the progress of lactation. Obtained results highlighted that an increase of the internal variation of the milk EC’ signal can be expected with the progress of lactation (since the values of amplitude of the peaks were generally higher in the case of the progress of lactation). Our research group found similar results in a previous study [28], analyzing the general statistical index: σ^2^_EC_. We found that σ^2^_EC_ increased during lactation stages for both healthy and infected glands. However, the results obtained in the present study allowed for a better description of the expected changes of the milk EC signal’s pattern during the progress of lactation. They show that EC signal’ pattern, when progressive lactation stages are evaluated, is generally characterized by slower fluctuations (due to the lower frequencies of the main peaks) and by a signal’s more irregular trend (due to the higher amplitudes of all the considered peaks).

The accuracy shown by the studied frequency peaks in the detection of the goat health status was not high when compared with results reported in other studies. Best values of specificity and sensitivity were reached by the frequency peak FFT_P_2_ (42.9% and 80.1%, respectively) using as parameter the amplitude of the peak and the frequency peak FFT_P_1_, (39.8% and 80.1%, respectively), using as parameter the frequency of the peak. However, these results were expected. Romero [29] reported low sensitivity and specificity of mastitis detection with EC, when different milking fractions and thresholds were evaluated. Furthermore, Romero [29] highlighted that such low performance was consistent with the results obtained by Díaz [33] in a study carried out to obtain further knowledge on milk EC as a tool for detecting mastitis in goats by analysing effects such as farm, parity, stage of lactation, SCC and health status. Also our research group found similar results. In a study on the use of time series evaluation of the milk EC to detect health status of dairy goats, and in another study that applied fuzzy logic technology to a multivariate model with the same aim, we confirmed that a better accuracy can be reached considering the intrinsic variation of animals and that simple thresholds have to be avoided (as in cows). However, all the studies on this topic suggest that a better knowledge of the relationship between the milk EC signal and the animal HS, in order to find more informative indexes, could lead to better results in detecting HS of dairy goats [26,27,28,29,32,33,34,35]. With this in mind, results obtained in the present study have to be evaluated. They showed that the studied frequency peaks were able to better characterize the milk EC signal than other traits. Therefore, they have to be considered a necessary step in order to find new indexes that should improve the performances of monitoring systems that evaluate the HS of dairy goats through the use of the milk EC in a multivariate approach.

Under a practical point of view, starting from these results, new milking systems for dairy goats could be developed. These systems could acquire the gland milk EC, online and during a milking, by sensors included in the improved milking clusters. At the end of milking, the Fourier frequency spectrum of the signal could be calculated and the frequency peaks determined. A following evaluation of acquired data, by a dedicated control algorithm, could allow the discrimination of the HS of each goat. If these monitoring systems will reach accuracies comparable with those obtained in dairy cows then positive results in terms of herd management will be achieved by farmers in the goat farming agricultural sector.

The future steps in this research will regard the developing of a complete monitoring system and a test of this system in a real scenario. The system will perform an automatic monitoring of the HS of goat using as input the data collected from each mammary gland by the EC sensors. The new indexes identified in the present study (but not only) will be used by the control algorithm in a multivariate approach. Different system set-ups will be tested in order to reach the best possible accuracy.

## 5. Conclusions

The present study showed that the Fourier frequency spectrum of the milk EC signal can be better characterized by the evaluation of the main frequency peaks and that the changes of milk EC signal patterns in the time domain can be well described by the changes of the corresponding frequency peaks. Furthermore, these indexes showed a significant relationship with the health status of the goat glands. Therefore, they could be useful in improving the performances of future monitoring systems—based on multivariate models—which evaluate the HS of dairy goats by the use of gland milk EC sensors.
